# A multiplex PCR strategy to screen for known mutations in families with sudden cardiac death burden

**DOI:** 10.14440/jbm.2017.181

**Published:** 2017-07-03

**Authors:** Giang Duong, Thomas M. Helms, Christoph A. Karle

**Affiliations:** 1 Medical Practice for Diagnostics, Hohenlohe–Künzelsau, Germany; 2 Department of Cardiology, Medical University Hospital, Heidelberg, Germany; 3 German Foundation for chronically ill, Hamburg, Fürth, Germany; 4 PeriCor Cardiology Working Group, Ass. UCSF, Hamburg, Germany

**Keywords:** sudden cardiac death, personalized medicine, point-of-care testing, PCR

## Abstract

Ventricular tachyarrythmia occurring in ischemic heart disease, dilated/hypertrophic cardiomyopathies or rare monogenic mutations of cardiac ion channels or associated proteins belong to the most frequent causes of sudden cardiac death (SCD). In further decades, next generation sequencing and bioinformatic analysis will become the gold standard of SCD risk stratification. At the moment, Sanger-sequencing is still obligatory in genetic diagnosis. A multiplex polymerase chain reaction (PCR) assay detecting eight SCD mutations in one reaction-tube was developed. To test the general validity of the assay, it was used with 12 patients, who had one or two of the eight mutations (LMNA, p.V256V; SCN5A, p.R1583C; RYR2, p.G1885E; MYH7, V606M; DSG2, p.T335A; KCNJ8, p.S422L; MYBPC, p.E441K; TNNT2, A38V). Thereafter, we tested the multiplex assay in a real diagnostic environment within a high risk family of several past SCD cases. This method allows efficient discrimination of multiple mutations by allele-specific PCR with standard PCR conditions. It relies on obtaining a PCR product specific to the mutation or wildtype—using primers that have the 3′end base complementary to the DNA template site, *i.e.* a specific primer only permits amplification to take place when its 3′terminal nucleotide matches with its target sequence. The PCR products are further analyzed by length, with Tape Station^®^(Agilent Technologies, Germany), a high-fidelity capillary chromatography test. The novel multiplex PCR assay strategy could be a good additional test used for SCD risk stratification. Advantages of the test are high velocity and ease of implementation, low price and flexibility of application within cardiomyopathy families for screening purposes.

## INTRODUCTION

Sudden cardiac death (SCD) is an unexpected fatal event due to cardiac disorders- accounting for 100000 to 200000 annual deaths in Germany [1].The most frequent cause of SCD is ventricular tachyarrhythmia occurring in ischemic heart disease (ca. 75%–80%); dilated cardiomyopathy (idiopathic and post-myocarditis, ca. 10%–15%) or rare monogenic mutations in cardiac ion channels or associated proteins (1%–2%). The mechanism of arrhythmia in cardiomyopathy might be explained by structural or geometrical changes of heart muscle although genetic or epigenetic changes of trigger proteins like ion channels might be another distinct cause of arrhythmia [2-4]. Molecular diagnostics and genetic testing could be helpful in these cases, since protein changes may have a genetic background and occur frequently in a familial setting. In further decades, next generation sequencing and bioinformatic analysis will become the gold standard of SCD risk stratification. At the moment Sanger-sequencing is still necessary for genetic diagnosis. Therefore, we developed a flexible multiplex test for some SCD causing mutations for family screening purpose after a genetic testing was made with a positive result. The other family members can be tested with the developed multiplex allele-specific polymerase chain reaction (AS-PCR).

The AS-PCR allows the detection of known SNPs in minimally equipped laboratories. Newton *et al*. were the first group to describe the AS-PCR technique for known point mutations in 1989 [5]. This method relies on the formation of a PCR product on the basis that the nucleotide at the 3′-end of the primer complements perfectly, meaning one primer is designed to mismatch one allele and match the other allele perfectly. Thus, one allele is preferably amplified. After Newton *et al*. [5] developed the AS-PCR, which permits the differentiation of any known mutation in genomic DNA, other innovative approaches were developed to detect specific PCR products. For example, “melting curve analysis” [6] which needs nucleic acid strains, or “hybridization by specifically labeled probes” [7]. AS-PCR is used in many areas of study such as the detection of genetic disorders [8-11], microbiology research [12], pharmacogenetics [13] and genotyping [14]. For every AS-PCR reaction, only one SNP can be examined.

In this work, we describe a new inexpensive multiplex AS-PCR method that allowed us to detect various known mutations, which are associated with SCD.

## MATERIALS AND METHODS

### Literature research and bioinformatics

**Figure 1.**
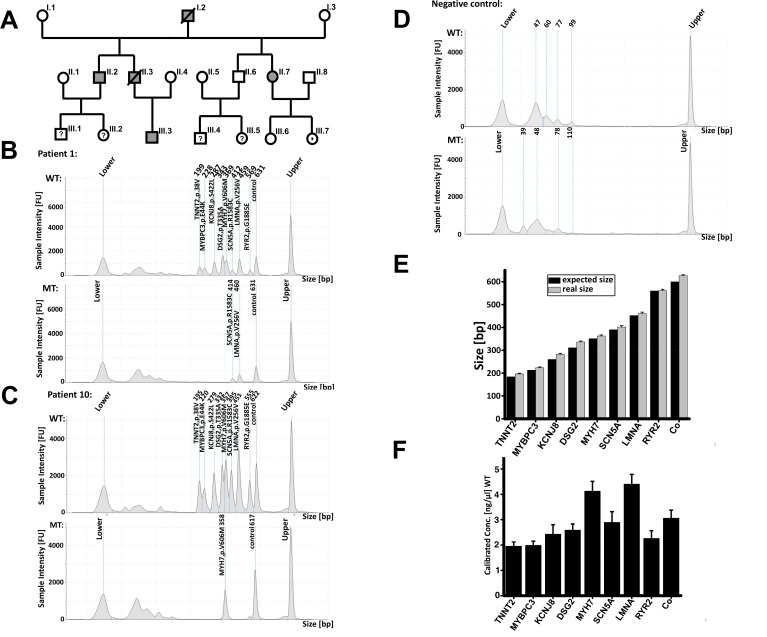
Pedigree and summary of the multiplex AS-PCR results. **A.** Family of the index patient (III.3). Uncle (II.2) and aunt (II.7) was diagnosed with dCMP and had to undergo heart transplantation. Father (II.3) (33 years) and grandfather (I.2) (56 years) died of SCD. **B.** The electropherogram profiles from patient one (III.3) from the dCMP and SCD family. Lower: lower marker; Upper: upper marker. **C.** Electropherogram from patient #10. **D.** Electropherogram from negative control. **E.** Summary of the product sizes of all twelve patients with SD of read lengths versus expected sizes. Error bars represent the standard deviation of all PCR products in the WT sample from all twelve patients. **F.** Summary of averaged concentrations from all PCR products from all twelve patients in the WT sample and the error bars represent the standard deviation.

Literature research was performed in PubMed under the category ClinVar with the key words “sudden cardiac death” and “pathogenic”. This database is a web-based overview of systemic meta-analysis and collective data and it is regularly updated with genetic association studies. The database listed 130 relevant mutations which are associated with SCD. Not all of them are real pathogenic; some of them are unclassified because of contradictory statements. For our purpose, it is fully adequate because our main focus is the development of a cost-efficient method for the diagnostic of familial SCD. This list was matched with a gene bank comprising many cases of SCD and cardiomyopathies (Benjamin Meder group, Cardiology, Internal Medicine III, University of Heidelberg). Within this gene bank, the DNA of 7 patients matched the criteria mentioned above found in the literature research. Five additional patients originated from our outpatient resources. The 5 patients are members of a huge family with a strong history of SCD (**Fig. 1A**). The index patient (III.3), a 28-year-old Caucasian male presented to our medical outpatient resource because of sharp pain in the breast. Echocardiography revealed normal left ventricular function but an ECG with atrioventricular block pattern (degree I). Because of a positive family-history of SCD, we advised him to undergo genetic testing. The genetic testing with NGS revealed two mutations: LMNA, c.768G>A and SCN5A, c.4747C>T. Overall we had 12 patients with one of the 130 mutations mentioned above (**Table 1**).

### Primer design

**Figure 2.**
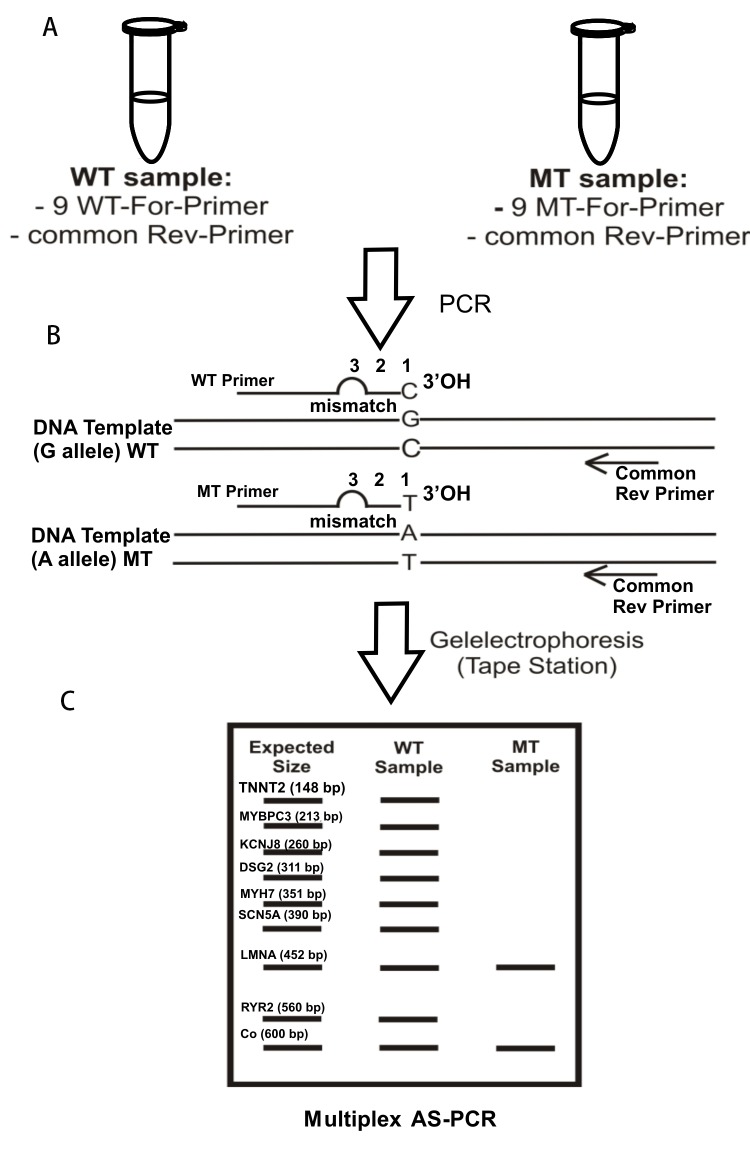
Schematic presentation of the multiplex AS-PCR method. **A.** Two reaction tubes were necessary for the assay. Content of the tubes: common reverse primer and different forward primer of all 8 + 1 control amplicons. The control primers (internal quality control) are the same primer pair in WT and MT tubes, taq-polymerase without 3′-5′exonuclease activity and template DNA. **B.** As an example, the mutation used here is a G->A substitution, but this method can be used for all other single base substitutions. Only a perfectly fitting forward primer guarantees an amplicon. This means: If the G allele is present, only the WT forward primer can bind to the DNA template and an amplicon, which presents the G allele, is generated. An A allele amplicon is generated when the MT forward primer can bind to the DNA. If the person is heterozygous at this position, two amplicons occur. Allele-specificity was increased by introducing a mismatch at position 2—counting from the 3′terminus. By positioning the reverse primer at different distances from the mutation, the nine amplicons differ in length, allowing them to be discriminated by gel electrophoresis. Only the control forward primer has no additional mismatches. The mutant specific nucleotide forms a base pairing only with the mutant allele. In contrast, the wildtype-specific nucleotide forms a base pairing only with the wildtype allele. **C.** After performing the multiplex PCR, products of both tubes are separated by Tape Station. Each of the nine amplicons was of a different length. Therefore, the mutations were detected on the basis of the patterns of their peak size.

Primers for the multiplex AS-PCR were designed using Primer3 web version 4.0.0 software and were scanned for uniqueness using NCBI BLAST search engine. The primer oligos were synthesized by Biomers (Germany, Ulm). For each mutation, the primer set included a reverse common primer (R) and two forward allele-specific primers (Fwt and Fmt), with the 3′ terminal base of each specific primer matching the wildtype or the mutant allele. The primers were designed to have a length of 19–28 nucleotides and to amplify fragments ranging from 180 to 600 bp and the fragments differ from each other between 30–62 bp. An unspecific mismatch was added to the allele-specific forward primer end to increase the allele specificity because only one mutation was insufficient to achieve the wanted level of discrimination. It is known that a single mismatch, at or near the 3′end can effect PCR more strongly than at other positions [14,15]. According to “Current Protocols in Human Genetics” [16], the strength of mismatch pairing are: “None”: AT, GC; “weak”: CA, GT; “medium”: AA, GG; “strong”: CC; “maximum”: GA, CT, TT (**Table 1**). As a general rule for the primer design, if the 3′end mismatch is a strong one, a weak secondary mismatch has to be incorporated. To distinguish between complete PCR failures from an informative no-amplification result, an internal control was added to WT and MT sample. The internal control for WT and MT sample consisted of the same primer pair with an amplicon length of 600 bp and without additional mismatch. The position of the primer pairs has to be chosen carefully—making sure that a different product length is to be formed for each mutation. Differentiation between the eight mutations and the internal control is by size length of amplification products. The principle of the new modified AS-PCR method is schematically presented in **Figure 2.** The primer pairs were at first used at the same concentration of 0.4 µM, but with equimolar primer concentrations uniform amplification signals for all fragments was not given therefore the concentration of some primer pairs were reduced or increased (**Table 1**).

### PCR

The Qiagen Multiplex PCR Plus Kit was used containing the HotStarTaq Plus DNA Polymerase without any 3′-5′exonuclease activity because using a DNA Polymerase with 3′-5′exonuclease activity shows false positive results (data not shown). Since the assay comprises two multiplex reactions, the primer sequence has to be designed with similar reaction kinetics. A C/G content of 40%–60% is suggested as general guideline for specific annealing. PCR for WT and MT tubes were carried out using a thermal cycler (Eppendorf Mastercycler, vapo.protect, Germany). Final volume of PCR reaction was 25 µl. The mixture of PCR comprised 1 × Multiplex PCR Master Mix (Qiagen, Germany), 2.5 µl Primer Mix, 150 ng DNA, 0.2 µl Taq polymerase. PCR was performed within 5 min of denaturation at 95°C, 35 cycles of 95°C for 30 s, annealing at 65°C for 90 s, 72°C for 30 s, and a final extension at 68°C for 10 min.

### Electrophoresis

After PCR, the products are separated and analyzed by electrophoresis with the Agilent 2200 TapeStation system (Agilent, Germany) with the High Sensitivity D1K Kit according to the manufacturer information and the band patterns should look like stairs (**Fig. 1E**).The method was validated by direct DNA sequencing or next generation sequencing.

For sanger sequencing, all mutated exons were amplified by polymerase chain reaction. After purification of the PCR products with magnetic bead-based AmPure XP system (Beckman Coulter), PCR fragments were sequenced in both directions using the GenomeLab™ DTCS Quick Start Kit and GeXP Genetic Analysis System (Beckman Coulter).

Next generation sequencing was performed on an Ion Torrent PGM (Life Technologies) according to information delivered by manufacturer. The study was conducted according to the recommendations of the Declaration of Helsinki, and was approved by the University of Heidelberg Human Ethics Committee.

## RESULTS

**Figure 1B** and **1C** shows the electropherograms of patient #1 (index patient; III.3), patient #10 (not from the index patient family) and negative control. WT samples from both patients showed nine peaks with the expected product sizes. The control peak at 600 bp was detected in the WT and MT sample. MT sample from patient #1 (index patient, III.3) showed two extra peaks with the sizes of 460 bp and 414 bp (**Fig. 1B**). This means that patient #1 had the LMNA and the SCN5A mutation, both mutations are heterozygous. Because of these findings, some other members of the family decided to make a genetic testing. We used our developed test to screen the members if they had these two specific mutations as well and the result was: Three members (II.2; II.7; III.7) had only the LMNA mutation. Two of them (II.2; II.7) had already a heart transplantation. The third member (III.7) with the LMNA mutation had to make echocardiography controls on an annual basis to enable to intervene more rapidly whenever anomalies are found (**Fig. 1A**). Seven additional patients with known mutations were tested with the developed assay. As an example, we just displayed one of the seven patients. MT sample of patient #10 (**Fig. 1C**) showed besides the control peak (617 bp) an additional peak with the size of 358 bp which represent the MYH7, *c.1816G>A* mutation, meaning that the mutation is heterozygous.

**Figure 3.**
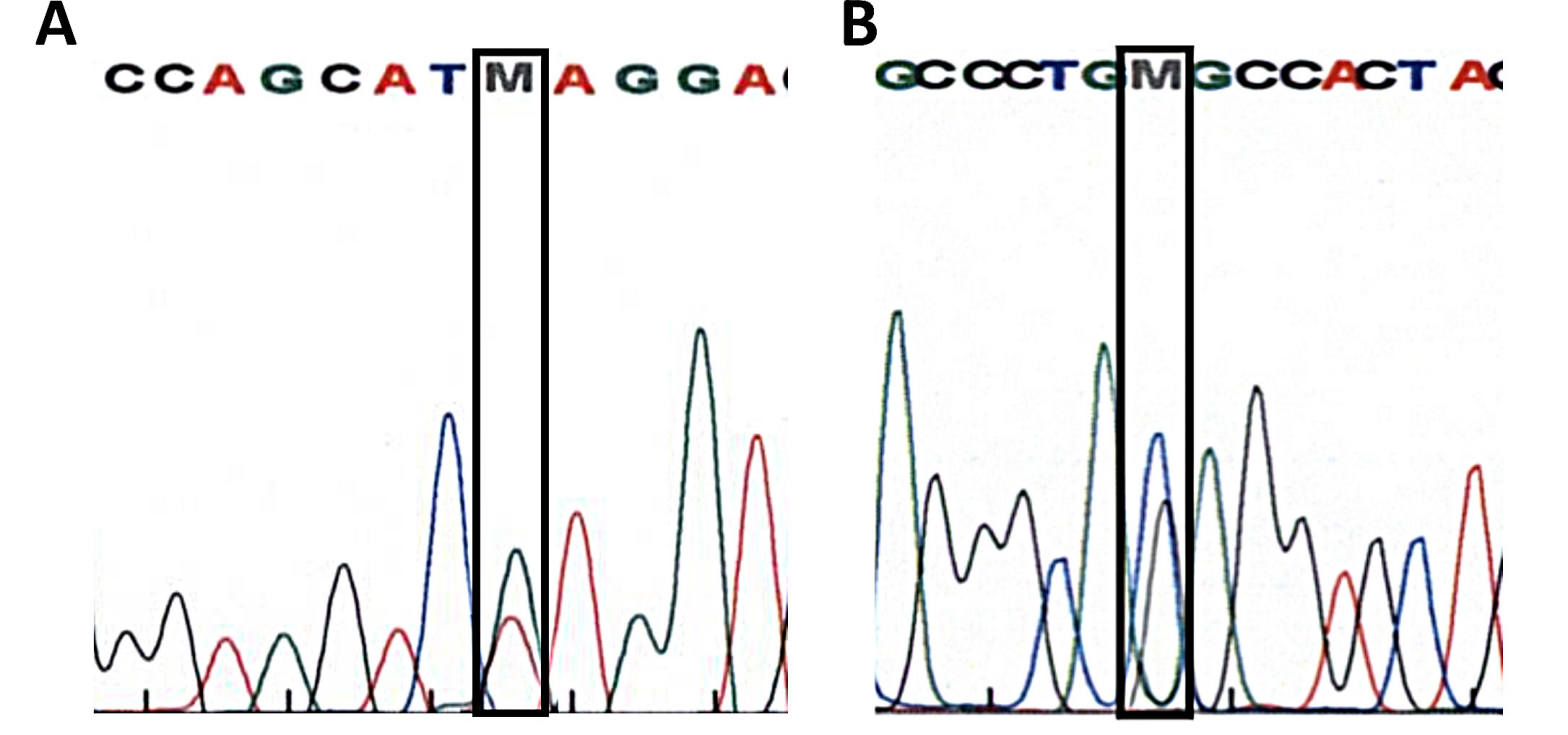
DNA sequencing results from patient 1(index patient) for LMNA, p.Val256Val (A) and SCN5A, p.Arg1583Cys (B).

In all 12 patients’ electropherograms, as well in the negative control (**Fig. 1D**) electropherograms, where the DNA was replaced with water, are undefined peaks in the region of 30–100 bp with a concentration higher than 0.4 ng/µl visible. A concentration higher than 0.4 ng/µl is the baseline for including in the analysis. In the region 200–700 bp where the expected products are located almost no background signals were visible thus showing the specificity of the primers (**Fig. 1D**). The unspecific peaks in the region of 30–100 bp had to be defined as artifacts, because the negative control electropherograms (**Fig. 1D**) showed the same unspecific peaks, although there was no DNA in the sample—deriving possibly from primer-primer interactions. The genotypes which were detected by help of this new multiplex AS-PCR assay were in 100% accordance with the sequencing results, **Figure 3** shows DNA sequences for LMNA, p.Val256Val and SCN5A, p.Arg1583Cys mutations from patient #1 elicited by Sanger technology as examples for the validation method.

To determine the sizing accuracy of the assay, PCR products from all 12 patients were analyzed and the corresponding sizes compared to the expected product sizes are summarized as a mean value and standard deviation was calculated (**Fig. 1E**). In general, the detected amplicons were 8–25 bp bigger than the excepted sizes but this did not distort the analysis because in the detected electropherograms all expected peaks are clearly separated from each other and above the background signal. Sizing precision was assessed by calculating the standard deviation (SD) from the size values obtained from all PCR-products in the WT samples of all twelve patients. The SD of the mean of amplicon size measurements was less than 6 bp, which demonstrates a stable determination of mutations and a valid determination of amplicon sizes.

Multiplex PCR with all primers produced the highest product concentration amounts of MYH7 and LMNA followed from SCN5A and control. All other amplicon concentration amounts were almost at the same concentration level. On the whole, the detected peak concentration of the MT samples was much lower compared to the WT samples. For example, in patient #10, the MYH7 peak in WT sample had a concentration of about 4.23 ng/µl and in the MT sample just 2.25 ng/µl but this did not disturb the analysis because the MT electrophero gram showed a clearly separated MYH7 peak without any background signals. Moreover, the concentration of this peak was higher than 0.4 ng/µl. All tests which were performed showed no false-positive results.

For peak formation, some factors are more critical than others: (1) Products smaller than 180 bp were not included because the smallest product expected was184 bp; (2) The concentration of the peaks must be higher than 0.4 ng/µl; and (3) Peaks should be clearly visible above the background signal.

Genetic analysis revealed heterozygous mutations of LMNA and SCN5A genes—same as with multiplex AS-PCR method in patient #1. Only the validation sequencing results of patient #1 are shown, as examples for all other patients. The LMNA gene allele included a substitution of G for A at position 768 (**Fig. 3A**) and the SCN5A gene allele included a substitution of C for T at position 4747 (**Fig. 3B**).

In the age of personalized medicine, fast, low-cost and reliable SNP detection methods attract increasing interests. In cardiovascular diseases, genetic testing and molecular diagnostics may be useful for preventive and therapeutic decisions in early stage of disease progression. In this way, family counseling will become much easier and better.

## DISCUSSION

At the moment Sanger-sequencing is the gold standard in genetic testing. Sanger-sequencing is very time consuming, so is next generation sequencing. In some cases, the patient has to wait months to get the results, and then the result is “no mutation” is found. Both methods are based on sequencing of whole gene areas. With our newly developed multiplex AS-PCR the simultaneous and rapid genetic testing of eight SCD variants is finished within three hours and the results are available within one day because only known mutations are tested, so the patient does not need to worry about long period delays of uncertainty. The multiplex AS-PCR can be used in family diagnostics, if one family member underwent a genetic testing and get a positive NGS result, the other family members can be analyzed with our developed test. In our case, the index patient (III.3) had two mutations: LMNA, p.V256V and SCN5A, p.R1583C.Therefore, we decided to insert an implantable cardioverter defibrillator (ICD), because of the incidence of two mutations and their high risk to get arrhythmia. The new assay combines two methods: multiplex PCR with AS-PCR. This improved method requires only two PCR tubes to differentiate between wildtype and mutant allele in eight different regions of DNA. An optimization procedure was implemented because equimolar primer concentrations and false annealing temperatures showed irregular fragment amplifications. Finally, a combination of different primer concentrations and an annealing temperature of 65°C achieved the expected sensitivity and specificity (**Table 1**).

The multiplex AS-PCR has the advantage of the minimal hands-on-effort and the handling is simple it can be developed to a point-of-care test (POC) with ready-to-use WT and MT samples. Hitherto, in the field of molecular genetics there are only a few POC tests available, because of the interpretation of the results and the many method steps. For prediction of sudden cardiac death burden, a “point-of-care test” is best of all possible test strategies for several reasons: (1) The test has to be fast in order to avoid cases of SCD during lab procedure that can take up to 3 months in some different settings; (2) The test has to be inexpensive because of the severity of consequences—it has to be accessible to all social layers independently from the insurance system—perhaps also for people in the third world countries; and (3) The diagnosis of sudden cardiac death burden hast to be based onto unequivocal research results—only some few mutational results might fit this condition, arising from former intensive bioinformatics. Other SNPs are unacceptable, since consequences for affected patients are severe from a psychological point of view, but also from putative treatments, *i.e.* implantation of heart rhythm devices.

With this study, we could establish a cost-effective, rapid and reproducible method, interesting for personalized medicine, as shown in our dCMP family. Thus the study is designed as a “proof-of-concept” study with the aim to show if a modified form of AS-PCR can detect known SCD mutations the case numbers can keep low.

The new multiplex AS-PCR assay could be a good additional test for SCD risk stratification for families with positive SCD history, but it is limited to only known mutations not excluding other mutations in other disease-causing genes.

To conclude, during past decades, a number of characteristics have been described to stratify cardiac patients to their risk of SCD. These tests address different cardiac (*e.g.*, pump function) and non-cardiac factors (*e.g.*, depression). The AS-PCR assay could serve as an additional test for familial SCD-burdened patients. Complex bioinformatics strategies are restricted to the initial design since the assay just detects well-known SCD mutations. Ease of implementation and further simplification of some steps within the test algorithm allow usage of the test as a standardized POC tool.

## Figures and Tables

**Table 1.** Mutations which are detectable with the multiplex AS-PCR*.

Gene	Mutation	Primer name	Primer sequence 5’-3’	Product size (bp)	Conditions	Concentration (µM)
TNNT2 (NM_000364.3)	c.113C>T (p.Ala38Val)	TNNT2_Fwt TNNT2_Fmt TNNT2_R	TCCAGAGCAGGAGGAGGCTGC TCCAGAGCAGGAGGAGGCTGT GAGTCAGGTGCACATGGGAAAGC	184	Primary familial hypertrophic cardiomyopathy Cardiomyopathy Increased left ventricular wall thickness	0.5
MYBPC3 (NM_000256.3)	c.1321G>A (p.Glu441Lys)	MYBPC3_Fwt MYBPC3_Fmt MYBPC3_R	CCAGTGCGTGGTGGGTGTCG CCAGTGCGTGGTGGGTGTCA CTTCACACTCAAACTCCACCCGC	213	Primary familial hypertrophic cardiomyopathy Cardiomyopathy	0.4
KCNJ8 (NM_004982.3)	c.1265C>T (p.Ser422Leu)	KCNJ8_Fwt KCNJ8_Fmt KCNJ8_R	CTCCAGAAGGAAATCAAAACACGTC CTCCAGAAGGAAATCAAAACACGTT TCACTGCGAATTCTACATGTATGA	260	Brugada syndrome	0.5
DSG2 (NM_001943.3)	c.1003A>G (p.Thr335Ala)	DSG2_Fwt DSG2_Fmt DSG2_R	GATGCTCAAACTAACGAAGGAATTGGGA GATGCTCAAACTAACGAAGGAATTGGGG TGCAAGAAAGCCTGGAAATGAGCT	311	Arrhythmogenic right ventricular cardiomyopathy Cardiomyopathy	0.4
MYH7 (NM_000257.3)	c.1816G>A (p.Val606Met)	MYH7_Fwt MYH7_Fmt MYH7_R	AGGATCCTCTCAATGAGACTGGCG AGGATCCTCTCAATGAGACTGGCA AAGAAGCATCAGTGTGGGGAGGT	351	Familial hypertrophic cardiomyopathy 1 Primary familial hypertrophic cardiomyopathy Cardiomyopathy	0.4
SCN5A (NM_198056.2)	c.4747C>T (p.Arg1583Cys)	SCN5A_Fwt SCN5A _Fmt SCN5A _R	GTCAAGCTGGCTGCCCGGC GTCAAGCTGGCTGCCCGGT CATCCTTGAAGCTCTCTAAGCAGTT	390	Brugada syndrome	0.3
LMNA (NM_170707.3)	c.768G>A (p.Val256Val)	LMNA_Fwt LMNA_Fmt LMNA_R	GCCCAGCATGAGGACCAGTTG GCCCAGCATGAGGACCAGTTA CATCCGGCCCAGACTCTAGG	452	Cardiomyopathy	0.3
RYR2 (NM_001035.2)	c.5654G>A (p.Gly1885Glu)	RYR2_Fwt RYR2_Fmt RYR2_R	GCTGGTGAGGAAGAAGCCAATGG GCTGGTGAGGAAGAAGCCAATGA CTGTAGAAGCTGAGGTGGGAGGA	560	Arrhythmogenic right ventricular cardiomyopathy	0.3
TNNT2 (NM_000364.3)	Ex 17	Control_F Control_R	TCCAGCTATCCCATTCCTCCTTG CGCCTCAGTTTCTCTCTCTCTCT	600	Control	0.3

*Precisely these eight different mutations were included to the test because only from these mutations patients' DNA were available (source: Orphanet, PubMed). Also the sequence of primers used for analysis, expected product sizes and concentrations.
